#  Survey of Accepted Practice following Failed Intubation for Emergency Caesarean Delivery

**DOI:** 10.1155/2015/192315

**Published:** 2015-03-10

**Authors:** Daniel Soltanifar, David Bogod, Sally Harrison, Brendan Carvalho, Pervez Sultan

**Affiliations:** ^1^Royal Free Hospital, Pond Street, London NW3 2QG, UK; ^2^Nottingham City Hospital, Hucknall Road, Nottingham NG5 1PB, UK; ^3^Stanford University School of Medicine, Stanford, CA 94305, USA; ^4^University College Hospital, 235 Euston Road, London NW1 2BU, UK

## Abstract

*Background*. There is no consensus on the optimum management of failed tracheal intubation in emergency cesarean delivery performed for fetal compromise. The decision making process on whether to wake the patient or continue anesthesia with a supraglottic airway device is an underexplored area. This survey explores perceptions and experiences of obstetric anesthetists managing failed intubation.* Methods*. Anesthetists attending the Group of Obstetric Anaesthetists London (GOAL) Meeting in April 2014 were surveyed.* Results*. Ninety-three percent of anesthetists surveyed would not always wake the patient in the event of failed intubation for emergency cesarean delivery performed for fetal compromise. The median (interquartile range) of perceived acceptability of continuing anesthesia with a well-fitting supraglottic airway device, assessed using a visual analogue scale (0–100; 0 completely unacceptable; 100 completely acceptable), was 90 [22.5]. Preoperative patient consent regarding the use of a supraglottic airway device for surgery in the event of failed intubation would affect the decision making of 40% of anaesthetists surveyed.* Conclusion*. These results demonstrate that a significant body of anesthetists with a subspecialty interest in obstetric anesthesia in the UK would not always wake up the patient and would continue with anesthesia and surgery with a supraglottic airway device in this setting.

## 1. Introduction

Fetal distress is the commonest reason for requiring general anesthesia (GA) for emergency caesarean delivery (CD). GA in this setting has an estimated failed intubation rate of 1 in 224 cases [1]. While reports of incidence and management strategies for failed intubation exist, there is little data exploring whether anesthetists would choose to continue emergency CD following failed intubation with a supraglottic airway if there is no immediate threat to the parturient's life [2, 3]. The United Kingdom Royal College of Obstetricians and Gynaecologists categorises the urgency of a CD into 4 grades. Category 1 CD is the one performed if there is immediate threat to the life of the woman or fetus. If the indication for CD is because of risk to maternal life, the decision is clearly in favour of continuing surgery without a definitive airway if ventilation is possible. However in the event of failed intubation during category 1 CD performed for fetal compromise, there appears to be no consensus regarding the optimal management strategy [4]. Waking the parturient will delay delivery of the fetus, which may potentially result in intrauterine death or neonatal hypoxic brain injury. This survey aimed to explore perceptions and experiences of anesthetists when managing failed intubation in the event of CD performed for fetal compromise.

## 2. Methods

An anonymous paper survey (see Appendix) was distributed to all delegates at the Group of Obstetric Anaesthetists London (GOAL) meeting held on 4th April 2014 at the Royal College of Anaesthetists, London. GOAL is an organisation which holds biannual meetings attended by anesthetists and aims to encourage obstetric anesthetic research and communication and exchange ideas within the London area. The survey questions were produced following a series of focused group discussions (authors DS, SH, and PS).

In addition to their grade of training, delegates were asked the following questions.

1. Have they ever personally experienced (or know a colleague who has experienced) failed intubation for category 1 CD without immediate threat to parturient's life? If so, how was the situation managed and were there any subsequent adverse events?
2. Would they* always* wake the patient up in the event of failed intubation for category 1 CD without immediate compromise to the parturient's life? What would influence decision making in this scenario? If they decided to proceed with surgery then what would be the preferred airway management strategy?
3. On a visual analogue scale (VAS) of 1–100 (completely unacceptable to completely acceptable) how acceptable would it be to continue surgery with a well-fitting supraglottic airway device in place for category 1 CD (without immediate threat to parturient's life) following failed intubation?
4. What advice would consultants give to a trainee in the event of failed intubation for category 1 CD for fetal distress now managed with a well-fitting supraglottic airway device in situ? The patient in the scenario is ASA 1, of normal BMI. The intubation was not possible with bougie and McCoy blade (Grade 4 laryngoscopy).
5. If preoperative consent was obtained from the patient regarding the use of a supraglottic airway device for surgery in event of an emergency would this change or affect decision making?

The survey results were collected in paper format during the meeting. The data was analysed using Microsoft Excel software (Version 2013, London). Demographic and outcome data are summarised with descriptive statistics and expressed as the median (interquartile range) and number (percentage) as appropriate.

## 3. Results

Of the 73 delegates that attended the meeting 60 anesthetists responded (response rate of 82%) of which 33 were consultants. Ninety-five percent of the respondents were either senior post-FRCA trainees, staff grades, associate specialists, or consultant grade (18%, 13%, 9%, and 55%, resp.). The remaining 5% were pre-FRCA level trainees (specialty training Grades 3-4).

Thirty percent of the delegates surveyed had personal experience in managing failed intubation for category 1 CD. The number of anesthetists from each grade experiencing failed intubation is summarised in Table 1. Approximately two-thirds (63%) of the delegates knew a colleague who had experienced failed intubation for category 1 CD delivery. The management strategies adopted by those who experienced failed intubation and those who knew colleagues who have managed this situation are summarised in Table 2. Of the anesthetists that had personally experienced failed intubation and continued with a supraglottic airway device, a quarter administered a neuromuscular blocking agent and only one reported an (unspecified) adverse outcome.

Ninety-three percent of the anesthetists surveyed would not always wake up the patient in the event of failed intubation for category 1 CD delivery. Factors determining the decision of whether or not to continue surgery with a supraglottic airway device are summarised in Table 3. Management strategies of the 93% of anesthetists that would consider continuing without a definitive airway are outlined in Table 4.

The median (IQR) VAS score for perceived acceptability of continuing category 1 CD with a well-fitting supraglottic airway device without immediate threat to parturient's life was 90 [22.5]. If preoperative consent was obtained from the parturient regarding the use of a supraglottic airway device for surgery in the event of failed intubation, this would change or affect 40% of anesthetists' decision making about whether to continue surgery without a definitive airway.

Finally, management strategies that consultants would give to trainees when asked for advice following failed intubation, now with a supraglottic airway device in situ, are outlined in Table 5.

## 4. Discussion

The main finding of this survey is that the majority of anesthetists surveyed regard continuing emergency CD with a supraglottic airway device (if there is no immediate threat to parturient's life) as acceptable practice.

This survey demonstrates that many of the anesthetists surveyed had directly been involved in or know colleagues that had been faced with this difficult scenario. Thirty percent of those surveyed had directly been involved in failed intubation scenario. This relatively high rate may be due to those surveyed having regular sessions in obstetrics increasing the likelihood of encountering failed intubation. Opinion on how to best manage this scenario may be changing with increasing number of reports describing safe supraglottic airway device use for large cohorts of elective GA cesarean deliveries [5–7]. This survey does suggest that many clinicians would continue anaesthesia and surgery with a well-fitting supraglottic airway device after failed intubation in an emergency. It is important to note that the survey questions did pertain to a patient with a normal BMI.

There are no studies to our knowledge examining long term maternal or fetal outcomes following the use of a supraglottic airway device to manage failed intubation. Although the Centre for Maternal and Child Enquiries (CMACE) report has not previously reported any poor outcomes with supraglottic airway devices, these airways are not routinely used for elective CD within the UK and we rely on case reports from emergency use which may provide an overly optimistic picture of their safety [8–10]. The decision to wake the parturient rather than continue surgery may result in intrauterine death and neonatal morbidity [11, 12]. Aspiration of blood or gastric contents still remains the most common cause of death after GA in modern obstetric anaesthetic practice, and 3 airway-related deaths were reported in the last triennial report [13, 14].

The majority of the 33 consultants surveyed would recommend administration of a neuromuscular blocking agent either with or without cricoid pressure (31% and 27%, resp.) with a supraglottic airway device in situ. Administering a neuromuscular blocking agent may have the advantage of ensuring adequate muscle relaxation to minimise coughing and straining and thus optimise conditions for effective surgery and airway management. Other authorities advocate avoidance of neuromuscular blockade in this setting and maintaining anesthesia utilizing a spontaneous breathing deep inhalational technique [15]. Use of the second generation laryngeal mask airway devices may be beneficial in this scenario and studies have previously described their successful use as a rescue airway device in obstetrics and use in elective caesarean deliveries [2, 7, 8]. Therefore if the decision to use a supraglottic airway device is made, there appears to be theoretical benefit in using a second generation supraglottic airway device with bite block and gastric port, for example, ProSeal laryngeal mask airway (Intavent Orthofix, Maidenhead, UK) or IGel (Intersurgical Inc., Berkshire, UK), Laryngeal Tube Suction, Combitube, and LMA Supreme (Teleflex, International) in order to minimise risk of aspiration [9, 10, 16]. Some authorities advocate the use of Combitube as it provides the advantage of some safety aspiration as well as providing the strongest fixation of the device, helping against accidental displacement of the supraglottic airway during the remainder of surgery [3, 17]. Indeed in one cadaver model the Combitube Easytube and Fastrach demonstrated the ability of withstanding increases in oesophageal pressure of up to 120 cm H_2_0 which would be advantageous in this setting particularly in the unfasted patient [18].

These findings of this survey may have an impact on future medicolegal cases involving claims for negligence. In England and Wales, the duty of care which must be fulfilled to avoid a finding of negligence, the Bolam principle, states that a practice would be deemed to be satisfactory if it was “accepted as proper by a responsible body of medical men skilled in that art” even if “there is a body of opinion that takes a contrary view” [19]. This rather flaccid test has been somewhat tightened up by the Bolitho rider, which demands that those supporting the practice in question must have taken into account “the comparative risks and benefits” and therefore reached a conclusion which is amenable to logical analysis [20].

This survey has shown that a significant body of anesthetists, faced with a patient who cannot be intubated while undergoing category 1 CD for fetal compromise, would allow surgery to continue with the airway supported by a supraglottic device. Following establishment of ventilation with a supraglottic airway the decision to proceed with surgery or awaken the patient involves weighing up of potential risks. In continuing with surgery there is the potential risk of maternal aspiration and loss of ventilation. Alternatively choosing to wake the patient runs the risk of complications associated with delayed delivery. This survey aimed to explore this difficult decision making. While the small size of the sample might be questioned, the survey result appears on the face of it to mean that this practice would pass the Bolam test. The decision to proceed is also logically sustainable and therefore Bolitho-supportable because it will minimise the risk of neonatal hypoxic-ischaemic injury, undoubtedly fulfilling a strong desire on the part of the mother and protecting her from psychological distress, and because the alternative, waking the mother up and proceeding to a regional block, is also not without maternal risk. It should be noted that Bolam specifically refutes the idea that there is only ever one acceptable action to take, and the anaesthetist choosing to wake the mother up would almost certainly also be protected against a claim of failure of duty of care on similar grounds, in this case the logical argument being that this approach might minimise risk to the mother, albeit at the cost of a unquantifiable risk to the neonate. An important ethical question arises from such cases, relating to whether it is acceptable to put an individual patient at increased risk (by continuing with a supraglottic device or, indeed, by using general rather than regional anaesthesia) in order to minimise risk to a second individual, the fetus, who at the time the decision is taken does not have a legal presence. Such a question is, however, a matter beyond the remit of this paper.

Informed consent could be obtained from the parturient prior to anaesthesia stating that, in the event of failed intubation, the parturient accepted increased risks of aspiration associated with supraglottic airway device, to improve the chances of successfully delivering a live neonate. Although this would influence the decision making process in 40% of surveyed anaesthetists, this would not be regarded by the majority of anaesthetists as a realistic option because the understanding of conduct of anaesthesia and its implications cannot be impressed to a parturient in the emergency situation and may result in undue distress for the parturient prior to emergency CD. Similarly, if the parturient was to make this decision antenatally this would not necessarily reflect their wishes at the time of labour and delivery and may not therefore be deemed as informed consent.

Limitations of this survey include the fact that only London anesthetists who attended the GOAL meeting were surveyed and the sample size is relatively small. The survey was formed from a focused group discussion of three anesthetists with an interest in obstetrics (DS, SH, and PS). There was however no trial or testing of questions prior to the survey being distributed so any potential ambiguity in the questions and responses should be considered. The survey results are also susceptible to nonresponder bias. The validity of VAS scoring in determining acceptability of continuing surgery with a supraglottic airway device in situ also requires further evaluation; however this was chosen to try to obtain a rating measure for the strength of the anesthetist's choice in the scenario.

This survey does not advocate continuing surgery with a supraglottic airway device following failed intubation for emergency CD for fetal compromise nor outline the optimal management in this situation. However these results do demonstrate that a significant body of anesthetists with a subspecialty interest in obstetric anesthesia in the UK would not always wake up the patient and would continue with anaesthesia and surgery with a supraglottic airway device in this setting. It also highlights that multiple factors are taken into consideration by anesthetists faced with making this difficult decision. Ultimately each case must be evaluated individually. Further studies are required to elucidate the safety of supraglottic airway devices in emergency CD as a rescue airway device in the obstetric population.

## Figures and Tables

**Table 1:** Number of respondents by grade who had experienced a failed intubation for category 1 caesarean delivery without immediate threat to parturient's life.

Grade of anesthetist	Number
ST 3-4	0
ST 5 or above	3
Staff grade	4
Associate specialist	1
Consultant	10

**Table 2:** Management strategies of delegates who have personally experienced failed intubation and know colleagues that have experienced the situation.

Management strategy	Individuals adopting strategy (*n* = 18)	Colleagues of individuals adopting strategy (*n* = 66)
Wake and perform regional anaesthesia	3 (17)	18 (27)
Secure airway with advanced technique before starting CS	2 (11)	2 (3)
Proceed with SAD for duration of CS	7 (39)	29 (44)
Proceed with SAD and cricoid pressure for duration of CS	6 (33)	5 (8)
Proceed with SAD until delivery and then secure airway with advanced technique	0	12 (18)

Values are presented as number (percentage).

CD: caesarean delivery; SAD: supraglottic airway device.

**Table 3:** Factors influencing the decision to continue caesarean delivery with supraglottic airway device, for anaesthetists that would consider continuing surgery.

Factor	Respondents (*n* = 56)
Comorbidities of parturient	31 (55)
BMI	39 (70)
Quality of SAD seal	42 (75)
Fasting status	24 (43)
Other	
Team skill	2 (4)
Clinical situation	2 (4)
Speed of surgeon	2 (4)

Values are presented as number (percentage).

BMI: body mass index; SAD: supraglottic airway device.

**Table 4:** Management strategies that anaesthetists would utilise if faced with failed intubation for category 1 caesarean delivery scenario (for anaesthetists that would consider continuing surgery).

Management strategy	Respondents (*n* = 56)
Proceed only after securing the airway with advanced techniques	2 (4)
Proceed with an SAD until delivery and then attempt to secure the airway with advanced techniques	24 (43)
Proceed with an SAD (and cricoid pressure) for the duration of case	27 (48)
Other	3 (5)

Values are presented as number (percentage).

SAD: supraglottic airway device.

**Table 5:** Management strategies consultants would advise trainees faced with a failed intubation for category 1 caesarean delivery, now with a supraglottic airway device in situ.

Management strategy	Advice given (*n* = 33)
Wake and perform regional	5 (15)
Administer muscle relaxant and continue surgery with SAD and cricoid pressure	10 (31)
Administer muscle relaxant and continue surgery with SAD for duration of case	9 (27)
Stop surgery, administer muscle relaxant, and secure airway with advanced techniques before proceeding	1 (3)
Proceed with SAD in place until delivery and then attempt to secure airway with advanced techniques	5 (15)
Other (depends on case specifics)	3 (9)

Values are presented as number (percentage).

SAD: supraglottic airway device.

## Appendix

### Survey

*Survey: Failed Intubation for Category 1 Caesarean Delivery for Fetal Distress*. We would like to know about your real-life experiences with managing failed intubation for category 1 caesarean delivery, specifically in the instance where there is no immediate danger to parturient's life but there are signs of fetal distress on CTG consistent with severe fetal compromise (prolonged sustained fetal bradycardia).

Grade
  CT1-2 □
  ST3-4 □
  ST5 or above □
  Staff grade □
  Associate Specialist □
  Consultant □
Q1. (A) Have you personally experienced failed intubation for category 1 CS without immediate threat to parturient's life?
  Y
  N
(B) If yes how many such cases have you directly been involved in?——
(C) How did you manage this/these cases (please enter number next to management strategy)?
  - Wake patient up and perform regional anaesthesia——
  - Wait to secure airway with advanced techniques, for example, asleep fibre optic intubation before starting CS——
  - Proceed with supraglottic airway device for duration of CS——
  - Proceed with supraglottic airway device and cricoid pressure for duration of CS——
  - Proceed with supraglottic airway device until delivery and then secure airway with advanced technique after delivery——
  - Other, please state——
(D) Were there any adverse outcomes if you have previously proceeded with supraglottic airway device for a CS?
  Y
  N
If yes please state what——
(E) When proceeding with supraglottic airway device did the patient receive nondepolarising neuromuscular blockade?
  Y
  N
Q2. (A) Do you know a colleague or trainee who has experienced failed intubation for category 1 CS (without compromise to parturient's life)?
  Y
  N
(B) How many of such cases do you know about that have occurred to your colleagues/trainees during your career?——
(C) How did they manage this/these cases (please enter number)?
  (I) Wake patient up and perform regional anaesthesia——
  (II) Wait to secure airway with advanced techniques, for example, asleep fibre optic intubation before starting CS——
  (III) Proceed with supraglottic airway device for duration of CS——
  (IV) Proceed with supraglottic airway device and cricoid pressure for duration of CS——
  (V) Proceed with supraglottic airway device until delivery and then secure airway with advanced technique after delivery——
  (VI) Other, please state——
(D) Were there any adverse incidents in cases continued on supraglottic airway device?
  Y
  N
Q3. (A) Would you personally* always* wake the patient up in the event of failed intubation for category 1 CS (for fetal distress without immediate compromise to the parturient's life)?
  Y
  N
(B) If no this would depend on
  Parturient's comorbidity □
  BMI □
  How well supraglottic airway device fits □
  Fasting status □
  Other, please state——
(C) If you consider continuing caesarean delivery without waking the parturient you would
  Proceed only after securing the airway with advanced techniques □
  Proceed with a supraglottic airway device until delivery and then attempt to secure the airway with advanced techniques □
  Proceed with a supraglottic airway device (and cricoid pressure) for the duration of case □
  Other——
Q4. On a scale of 1–100 how acceptable do you feel it is to proceed with a well-fitting supraglottic airway device in place for category 1 CS with threat to fetal life (without immediate threat to parturient's life) following failed intubation in a woman with normal BMI and no past medical history? Please place a line on the scale.
0–––––––––––––––––-100
Completely unacceptable Completely acceptable
Q5. If preoperative consent was obtained from the patient on the use of a supraglottic airway device for surgery in event of an emergency would this change or affect your decision making?
  Y
  N
Comments——
Q6. For consultants only:
What advice would you give to a post-FRCA trainee over the phone in the event of them calling you about failed intubation for category 1 CS for fetal distress? They say that the patient is otherwise fit and well and the intubation was not possible with bougie and McCoy blade (Grade 4 laryngoscopy) after 2 attempts but the supraglottic airway device is well-fitting and parturient is of normal BMI and cricoid pressure is being applied.
Wake the patient up and perform regional anaesthesia □
Administer muscle relaxant and continue surgery on supraglottic airway device with cricoid pressure □
Administer muscle relaxant and continue surgery on supraglottic airway device for duration of case □
Stop surgery, administer muscle relaxant, and secure airway with advanced techniques before proceeding □
Proceed with supraglottic airway device in place until delivery and then attempt to secure airway with advanced techniques □
Other, please state——
